# Comparative efficacy of BG-Sentinel 2 and CDC-like mosquito traps for monitoring potential malaria vectors in Europe

**DOI:** 10.1186/s13071-022-05285-9

**Published:** 2022-05-07

**Authors:** Michela Bertola, Diletta Fornasiero, Sofia Sgubin, Luca Mazzon, Marco Pombi, Fabrizio Montarsi

**Affiliations:** 1 Istituto Zooprofilattico Sperimentale delle Venezie, 35020 Legnaro, Padua Italy; 2 Department of Public Health and Infectious Diseases, Sapienza University of Rome, Rome, Italy; 3 Department of Agronomy, Food, Natural Resources, Animals and Environment (DAFNAE), University of Padova, 35020 Legnaro, Padua Italy

**Keywords:** *Anophelesdaciae*, *Anophelesmesseae*, *Anophelesmaculipennis* sensu stricto, BG-Sentinel trap, Centers for Disease Control and Prevention light trap, Italy

## Abstract

**Background:**

Different trapping devices and attractants are used in the mosquito surveillance programs currently running in Europe. Most of these devices target vector species belonging to the genera *Culex* or *Aedes*, and no studies have yet evaluated the effectiveness of different trapping devices for the specific targeting of *Anopheles* mosquito species, which are potential vectors of malaria in Europe. This study aims to fill this gap in knowledge by comparing the performance of trapping methods that are commonly used in European mosquito surveillance programs for *Culex* and *Aedes* for the specific collection of adults of species of the *Anopheles maculipennis* complex.

**Methods:**

The following combinations of traps and attractants were used: (i) BG-Sentinel 2 (BG trap) baited with a BG-Lure cartridge (BG + lure), (ii) BG trap baited with a BG-Lure cartridge and CO_2_ (BG + lure + CO_2_), (iii) Centers for Disease Control and Prevention-like trap (CDC trap) baited with CO_2_ (CDC + CO_2_), (iv) CDC trap used with light and baited with BG-Lure and CO_2_ (CDC light + lure + CO_2_). These combinations were compared in the field using a 4 × 4 Latin square study design. The trial was conducted in two sites in northeastern Italy in 2019. *Anopheles* species were identified morphologically and a sub-sample of *An. maculipennis* complex specimens were identified to species level by molecular analysis.

**Results:**

Forty-eight collections were performed on 12 different trapping days at each site, and a total of 1721 *An. maculipennis* complex specimens were captured. The molecular analysis of a sub-sample comprising 254 specimens identified both *Anopheles messeae/Anopheles daciae* (*n* = 103) and *Anopheles maculipennis* sensu stricto (*n* = 8) at site 1, while at site 2 only *An. messeae/An. daciae* (*n* = 143) was found. The four trapping devices differed with respect to the number of *An. messeae*/*An. daciae* captured. More mosquitoes were caught by the BG trap when it was used with additional lures (i.e. BG + lure + CO_2_) than without the attractant, CO_2_ [ratio_BG+lure vs BG+lure+CO2_ = 0.206, 95% confidence interval (CI) 0.101–0.420, *P* < 0.0001], while no significant differences were observed between CDC + CO_2_ and CDC light + lure + CO_2_ (*P* = 0.321). The addition of CO_2_ to BG + lure increased the ability of this combination to capture *An. messeae/An. daciae* by a factor of 4.85, and it also trapped more mosquitoes of other, non-target species (*Culex pipiens*, ratio_BG+lure vs BG+lure+CO2_ = 0.119, 95% CI 0.056–0.250, *P* < 0.0001; *Ochlerotatus caspius*, ratio_BG+lure vs BG+lure+CO2_ = 0.035, 95% CI 0.015–0.080, *P* < 0.0001).

**Conclusions:**

Our results show that both the BG-Sentinel and CDC trap can be used to effectively sample *An. messeae/An. daciae*, but that the combination of the BG-Sentinel trap with the BG-Lure and CO_2_ was the most effective means of achieving this. BG + lure + CO_2_ is considered the best combination for the routine monitoring of host-seeking *An. maculipennis* complex species such as *An. messeae/An. daciae*. The BG-Sentinel and CDC traps have value as alternative methods to human landing catches and manual aspiration for the standardized monitoring of *Anopheles* species in Europe.

**Graphical abstract:**

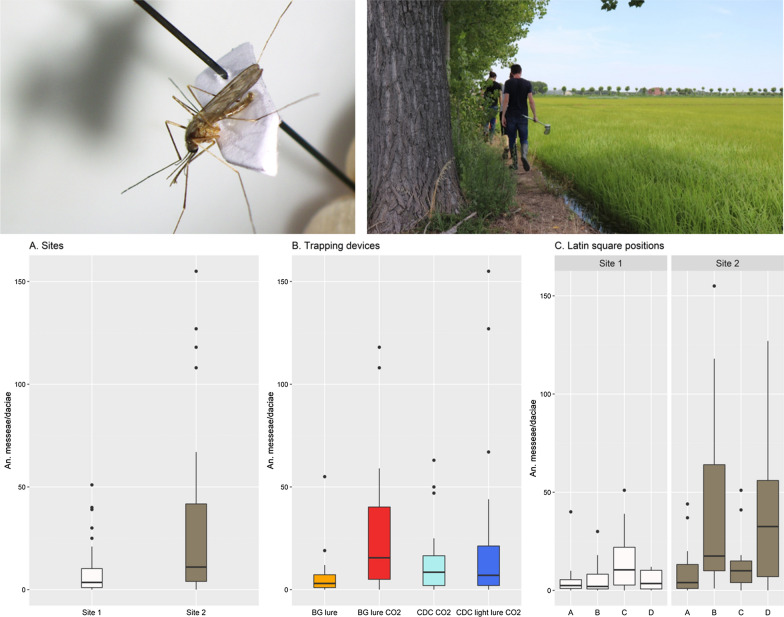

**Supplementary Information:**

The online version contains supplementary material available at 10.1186/s13071-022-05285-9.

## Background

Europe has been considered malaria-free by the World Health Organization since 1975, with the interruption of indigenous malaria transmission since that time [1]. Although eradication programs successfully arrested the circulation of malaria in Europe, they failed to eliminate the competent vectors, *Anopheles* species belonging mainly to the *Anopheles maculipennis* complex. Several *Anopheles* species competent for malaria transmission are still present throughout Europe [2–4]. The most common and widely distributed species belong to the *An. maculipennis* complex, which includes several species with morphologically indistinguishable adults. Molecular identification of the species of this complex is paramount because they do not have the same susceptibility to different *Plasmodium* species [5–7].

Following the eradication of malaria in almost all European countries, and due to the limited incidence of the disease in Europe since the 1970s, scientific interest in the *An. maculipennis* complex has progressively decreased. Consequently, in the last decades, only a few studies assessing the distribution of the European anopheline fauna have been carried out. However, there are still areas with residual malariogenic potential in various European countries, and in Italy in particular [6, 8], and several cases of cryptic malaria were recorded from 2000 to 2018 [9]. In this regard, it is important to monitor and provide regular updates on the presence and distribution of potential malaria vectors through the implementation of systematic and harmonized surveillance programs throughout Europe to promptly identify potential indigenous transmission events.

A recent study gives an update on the current distribution of the *An. maculipennis* complex in northern Italy, thanks to extensive field sampling and species identification [10]. However, *Anopheles* mosquitoes were collected mainly by Centers for Disease Control and Prevention (CDC) traps baited with CO_2_ in that study, which was undertaken in the context of entomological monitoring for West Nile Virus surveillance [11]. Typically, mosquito monitoring and surveillance programs presently underway in many European countries have been devised to target different vectors of disease within the genera *Culex* or *Aedes*. These programs are intended to monitor adult female mosquitoes (host-seeking, ovipositing or resting) by using different types of trapping devices and various methods of mosquito attractants (e.g. CO_2_, heat, olfactory lures, or light) [12].

The choice of sampling method depends on the target mosquito species and the study goal [13–15]. For example, to obtain accurate data on a mosquito population in a given area, the most suitable sampling methods must be chosen in relation to the behavior of the target mosquito species.

In areas where malaria is endemic, major vectors are often anthropophilic, and the use of human landing catches (HLC) remains the most direct and reliable method for evaluating mosquito density, mosquito biting activity and entomological inoculation rate [13, 16–19]. However, HLC have underlying ethical issues and involve the risk that human volunteers will come into contact with potentially infected vectors [20]. To avoid human contact with mosquitoes, efficient and reliable alternative tools have been developed to monitor and study vectors in areas endemic for malaria [21–26].

However, to the best of our knowledge, no European studies have evaluated the effectiveness of different trapping devices that specifically target *Anopheles* mosquito species. According to the literature, the method most frequently used to catch *Anopheles* mosquitoes in Europe is manual aspiration of resting adults in indoor shelters [15]. For the *An. maculipennis* complex, the CDC light trap seems to be an effective device [27], although for anthropophilic species HLC remain the gold standard [15, 28]; other sampling approaches have been poorly investigated for this complex.

The aim of the present study was to fill this gap in knowledge as follows: (i) compare the sampling performance of different trapping methods (CDC-like and BG-Sentinel traps baited with different combinations of lures and CO_2_) commonly used in European mosquito surveillance programs to specifically collect adult of the *An. maculipennis* complex species, (ii) evaluate the influence of the presence of hosts on the effectiveness of the trapping methods, (iii) assess malaria vector presence/absence and their abundance in sampled sites in order to update the Italian data, (iv) compare the efficacies of the tested traps for the capture of other abundant mosquito species (*Culex pipiens* and *Ochlerotatus caspius*).

## Methods

### Study area and experimental design

**Fig. 1:**
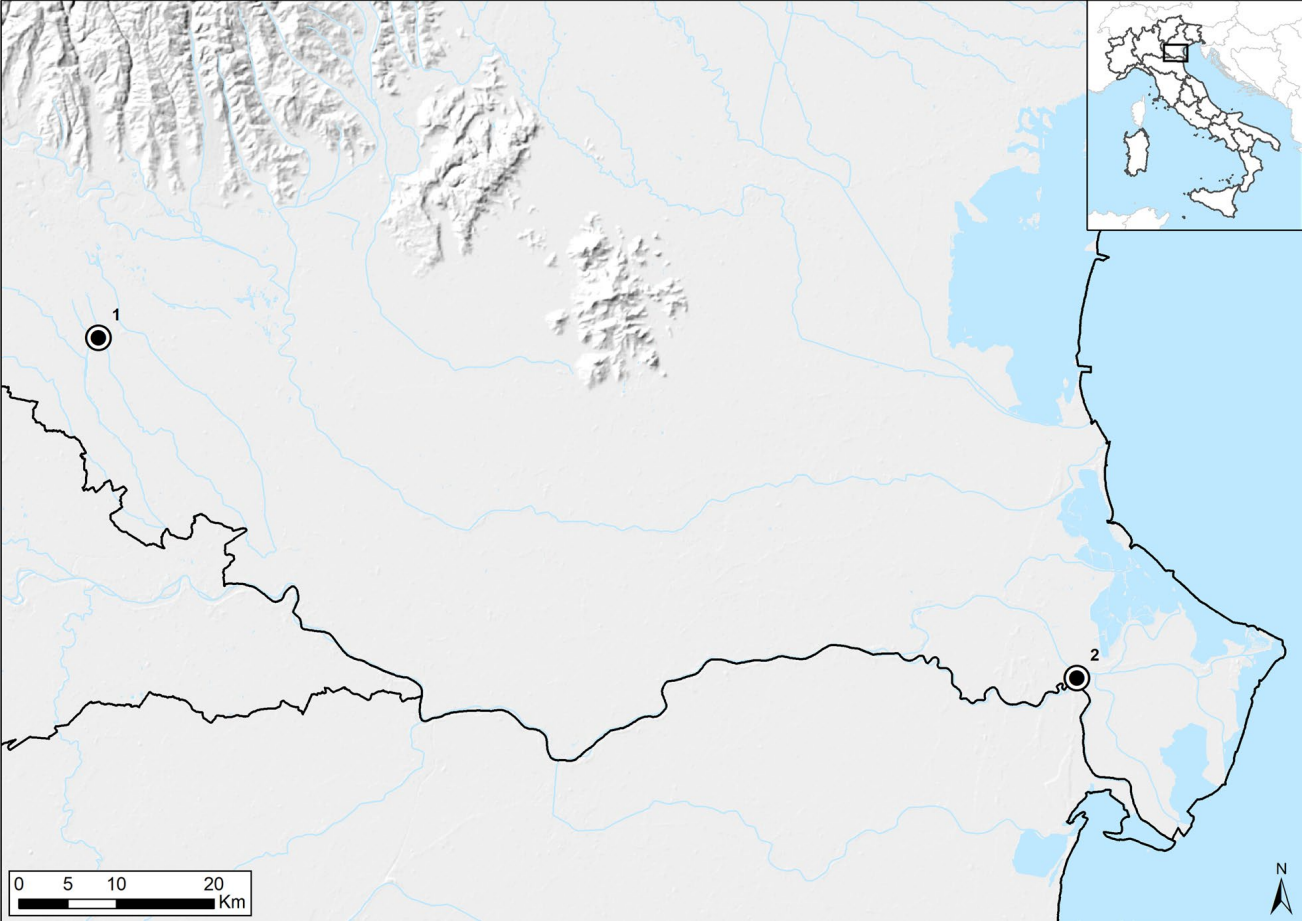
Location of the study area. Points represent sampling sites 1 (farm) and 2 (abandoned house) (maps made using ArcGIS Desktop; Release 10.5.1; Environmental Systems Research Institute, Redlands, CA; copyright 1999–2017)

An indication of the most suitable sites for *Anopheles* mosquitoes in northern Italy was given by the results of a previous study [10]. Based on this, two sites located in the Veneto Region (northeastern Italy) were selected for the present study, in accordance with the following criteria: countryside location, high abundance of *Anopheles* mosquitoes based on historical data, and the presence of wild/farmed animals. Two ecologically similar sampling sites, both surrounded by paddy fields, with the same elevation and climatic dynamics, but with a different density of farmed animals, were chosen to evaluate the possible influence of potential hosts in close proximity to the traps on captures. The first site was located on a farm (site 1; Verona Province; 45.29055N, 11.018483E), where 22 different animal species were kept (horses, goats, and chickens were among the most abundant species); the second site was an abandoned house in a rural area without farmed animals (site 2; Rovigo Province; 44.947222N, 12.279425E) (Fig. 1). At the farm, human presence was limited almost exclusively to the daytime, and only one person remained on site at night.

A preliminary single aspiration was performed at each site in summer 2018 to confirm *Anopheles* mosquito presence. Resting mosquitoes were collected indoors by sweeping a CDC Back Pack aspirator (model 1412; John W. Hock, FL) over the walls and ceilings of animal shelters at site 1, and in the abandoned house at site 2 for a 3-min period. Field-collected mosquitoes were stored and identified according to the same methodology described below. In total, 1059 specimens of *An. maculipennis* complex were collected: 955 at site 1 (farm) and 104 at site 2 (abandoned house). A sub-sample of the collected mosquitoes (*n* = 160, 16.8% at site 1 and* n* = 24, 23.1% at site 2) were identified using molecular methods, of which 147 (91.9%) were *Anopheles messeae/Anopheles daciae* and 13 (8.1%) *Anopheles maculipennis* sensu stricto (s.s.) at site 1, while 23 (95.8%) were *An. messeae/An. daciae* and one (4.2%) was *An. maculipennis* s.s. at site 2.

Trap comparisons were carried out in 2019 from July to September during the period of peak mosquito density (as assessed in previous samplings). Three replicates and 12 total days of collection were conducted at each site (Additional file 1: Table S1) using two different traps with different combinations of three attractants. The two traps used in the test were the BG-Sentinel 2 Mosquito Trap (BG trap; Biogents, Regensburg, Germany), and a Centers for Disease Control and Prevention-like trap [CDC trap; Italian Mosquito Trap (IMT); PeP, Cantu, Italy]. These traps were designed to collect host-seeking mosquitoes by aspiration, but they differ in their mechanisms of attraction and trapping. Both visual and olfactory attractants can be used with these traps, as follows: light (only with the CDC trap), CO_2_ provided by dry ice, and a BG-Lure cartridge (Biogents). The following combination of traps and attractants were used: (i) BG trap with BG-Lure cartridge (BG + lure), (ii) BG trap with BG-Lure cartridge and CO_2_ (BG + lure + CO_2_), (iii) CDC trap baited with CO_2_ (CDC + CO_2_), (iv) CDC trap with light and baited with BG-Lure cartridge and CO_2_ (CDC light + lure + CO_2_).

CDC traps were hung on low trees or wooden posts (trap opening at approximately 1-m height from the ground) outdoors. When used with the CDC traps, the lure cartridge and light were fixed near the suction fan of the trap. BG traps were placed on the ground following the manufacturer’s instructions, with the trap opening at 40-cm height from the ground. To use CO_2_ with the BG trap, the same type of thermic cartridge as that used with the CDC trap was placed 20 cm above the trap and filled daily with dry ice. The trap comparison experiment was set up as a 4 × 4 Latin square. At each location, all the traps were placed approximately 50 m from each other at four different sampling points. To eliminate any position-specific effect, all the traps were rotated to the next position every 24 h four times during the trapping cycle, so that each trap occupied every position during the cycle. Every 24 h, in the late afternoon, mosquitoes from each trap were collected, transported in dry ice, and stored at − 20 °C until processed in the laboratory. After mosquito collection, traps were rotated and refilled with dry ice. The same lure cartridges were used for each trapping cycle and were changed for new ones for the following cycle.

### Mosquito identification

Mosquitoes were morphologically identified under a stereomicroscope using taxonomic keys [29]. A representative sub-sample of specimens belonging to the *An. maculipennis* complex, which had been caught in 2018 by aspiration and in 2019 by trapping, was identified to species level by molecular methods. The sub-samples were randomly chosen from specimens captured every day by each trap at each site (sample) during the whole trapping period up to a maximum of 13 specimens per sample.

Using just one leg from each specimen, DNA was extracted following the animal tissue dilution and storage protocol of the Phire Tissue Direct PCR Master Mix (ThermoFisher Scientific, Waltham, MA). The extracts were analyzed as described in Lühken et al. [30]. Briefly, the assay is a species-specific multiplex quantitative PCR targeting the internal transcribed spacer 2 of ribosomal DNA. It is based on two primer sequences conserved among the species *Anopheles atroparvus*, *Anopheles maculipennis* s.s. and *Anopheles messeae* sensu lato (s.l.), and two different TaqMan probes for the latter species.

As the splitting of *An. messeae* into the two species *An. messeae* and *An. daciae* [31, 32] is not universally accepted, we overcame this controversial issue by adopting the definition *An. messeae/An. daciae*, without discriminating between the two taxonomic units.

### Meteorological and mosquito data

Meteorological data were obtained from two meteorological stations both approximately 7 km from the study sites. Total daily precipitation (Prec.; millimeters) and mean daily temperature (Tmean; degrees Celsius) during the sampling periods were extracted from the web sites of the Agenzia Regionale per la Prevenzione e Protezione Ambientale del Veneto [33].

For *Anopheles* collected in 2019, the proportions of *An. messeae*/*An. daciae* and *An. maculipennis* s.s. identified by molecular analysis were compared to the total *An. maculipennis* s.l. collected by all the traps and the *An. messeae*/*An. daciae* proportion calculated. As a result, the statistical analyses (preliminary tests and model building) were performed entirely for *An. messeae/An. daciae*, which represented almost all of the *Anopheles* specimens captured. The final model was then applied to the two other most abundant species of the sampled mosquito population (i.e. *Cx. pipiens* and *Oc. caspius*) to obtain a more complete overview of the trapping efficacies of the devices.

### Statistical analysis

The efficacy, defined as the number of trapped mosquitoes, of the four different trapping devices was evaluated by a series of statistical tests and a linear model. The first step consisted of univariate analyses to (i) evaluate the devices’ trapping capabilities in catching mosquitoes (Kruskal–Wallis rank sum test), (ii) assess the effect of the capture sites on the number of trapped mosquitoes (Wilcoxon test), (iii) test the trap position effect within the Latin square on captured *An. messeae*/*An. daciae* abundance (Kruskal–Wallis rank sum test), and (iv) assess the effects of daily precipitation and mean temperature on the number of trapped *An. messeae*/*An. daciae* (univariate linear models). The likelihood ratio test was then used to identify the best final model through the comparison of different nested models built by sequentially adding one or more variables of interest. A generalized linear model (GLM) with a negative binomial error distribution was chosen to model the count of trapped *An. messeae*/*An. daciae*, so that overdispersion, which is frequently associated with non-transformed richness data, could be accounted for [34]. The choice of a negative binomial over a Poisson regression was assessed with the help of diagnostic plots and dispersion tests (Additional file 2: Fig. S1). The parameter θ, which can be interpreted as a measure of data dispersion in the calculation of the variance of the negative binomial distribution, was estimated by means of maximum likelihood [35]. In order to obtain a direct estimate of the two categorical independent variables included in the regression, we omitted the estimation of the intercept to allow the model to reparameterize the remaining categorical covariates. Thus, the interpretation of the coefficient estimates could be done directly, as they did not represent the difference between each group and the reference group (i.e. the intercept). As the negative binomial regression uses a log–link function to link the linear combination of the predictors, the coefficients estimates must be interpreted with an additive effect on the log(*y*) scale, and with a multiplicative effect on the* y* scale (i.e. when the coefficients are back-transformed through the exponential function). Finally, contrasts among estimated marginal means were computed to evaluate the significant differences among the tested trapping devices, considering a confidence level of 0.95. The final GLM was also done for the two most abundant mosquito species (*Cx. pipiens* and *Oc. caspius*) in the sampled population.

All data cleaning and preparation, statistical analyses and model building were conducted using R statistical software version 4.0.5 [36] and the packages ggplot2 [37], MASS [35], emmeans [38] and DHARMa [39].

## Results

A total of 48 captures were performed for each site on 12 different days of collection in 2019, from 9 July to 6 September at site 1 (farm) and from 2 July to 30 August at site 2 (abandoned house) (Additional file 1: Table S1).

Overall, 25,442 mosquitoes belonging to 10 species were caught: 11,514 specimens at site 1, and 13,928 at site 2 (Table 1). *An. maculipennis* s.l. was the third most abundant taxon collected at both sites, representing 6.8% of trapped mosquitoes. Overall, 1721 specimens of *An. maculipennis* s.l. were collected, 437 (3.8%) at site 1 (farm) and 1284 (9.2%) at site 2 (abandoned house). A sub-sample of 254 specimens [*n* = 111 (25.2%) at site 1 and *n* = 143 (11.1%) at site 2] were identified to species level by molecular analysis. At site 1 (farm), both *An. messeae*/*An. daciae* (*n* = 103) and *An. maculipennis* s.s. (*n* = 8) were captured, while at site 2 (abandoned house), only *An. messeae*/*An. daciae* was found (*n* = 143) (Additional file 1: Table S2).

**Table 1:** Total number of adults of mosquito species collected at site 1 (farm) and site 2 (abandoned house) (percentages are shown in parentheses) in 2019 by the four different trapping devices^a^

	Site 1						Site 2				
	BG + lure + CO_2_	BG + lure	CDC light + lure + CO_2_	CDC + CO_2_	Total no. of mosquitoes		BG + lure + CO_2_	BG + lure	CDC light + lure + CO_2_	CDC + CO_2_	Total no. of mosquitoes
*Culex pipiens*	2457 (31.0)	249 (3.1)	1710 (21.6)	3515 (44.3)	7931		936 (11.6)	146 (1.8)	2494 (30.9)	4504 (55.7)	8080
*Ochlerotatus caspius*	1178 (41.4)	11 (0.4)	1267 (44.5)	391 (13.7)	2847		1288 (30.8)	105 (2.5)	2111 (50.4)	683 (16.3)	4187
*Anopheles maculipennis* sensu lato^b^	241 (55.1)	37 (8.5)	77 (17.6)	82 (18.8)	437		439 (34.2)	117 (9.1)	484 (37.7)	244 (19.0)	1284
*Aedes vexans*	0	1 (100.0)	0	0	1		10 (6.7)	1 (0.7)	80 (53.7)	58 (38.9)	149
*Aedes albopictus*	40 (48.2)	35 (42.2)	4 (4.8)	4 (4.8)	83		14 (34.1)	21 (51.2)	0	6 (14.6)	41
*Culex modestus*	2 (6.5)	0	12 (38.7)	17 (54.8)	31		1 (10.0)	1 (10.0)	3 (30.0)	5 (50.0)	10
*Coquillettidia richardii*	0	0	1 (25.0)	3 (75.0)	4		0	0	0	2 (100.0)	2
*Anopheles plumbeus*	0	0	0	0	0		0	0	0	2 (100.0)	2
*Culiseta annulata*	1 (100.0)	0	0	0	1		0	0	0	0	0
ND	5 (2.8)	1 (0.6)	25 (14.0)	148 (82.7)	179		0	0	25 (14.5)	148 (85.5)	173
Total	3924 (34.1)	334 (2.9)	3096 (26.9)	4160 (36.1)		11,514	2688 (19.3)	391 (2.8)	5197 (37.3)	5652 (40.6)	13,928

*ND* Species not determined

^a^BG-Sentinel 2 (BG trap) baited with a BG-Lure cartridge and CO_2_ (*BG + lure + CO*_2_), BG trap baited with a BG-Lure cartridge (*BG + lure*), Centers for Disease Control and Prevention-like trap (CDC) used with light and baited with BG-Lure and CO_2_ (*CDC light + lure + CO*_2_), CDC baited with CO_2_ (*CDC + CO*_2_)

^b^*Anopheles messeae/Anopheles daciae* and/or *Anopheles maculipennis* sensu stricto

**Fig. 2:**
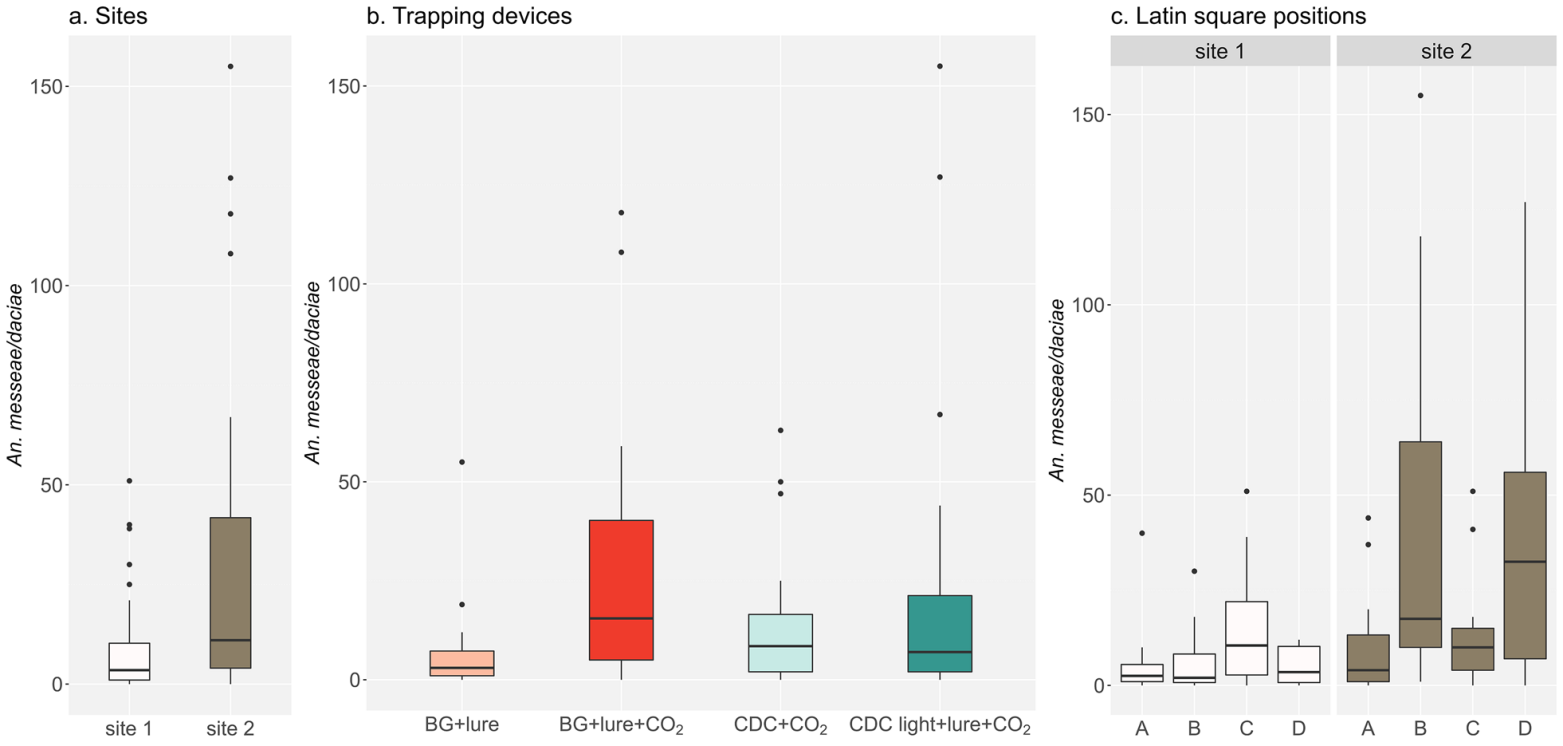
Box plots of *Anopheles messeae/Anopheles daciae* abundance distributions for each study site (**a**), type of trapping device (**b**), and Latin square position (**c**) for site 1 (farm) and site 2 (abandoned house). The middle, lower and upper hinges of the box plots represent the 50%, 25% and 75% quantiles, respectively. *BG lure* BG-Sentinel 2 (BG trap) baited with a BG-Lure cartridge, *BG lure+CO*_2_ BG trap baited with a BG-Lure cartridge and CO_2_, *CDC+CO*_2_ Centers for Disease Control and Prevention-like trap (CDC) baited with CO_2_, *CDC light+lure+CO*_2_ CDC trap used with light and baited with BG-Lure and CO_2_

To assess the effects of the type of device, trapping site and Latin square location on the total number of trapped *An. messeae*/*An. daciae*, a series of non-parametric statistical tests was performed, following a preliminary analyses of data normality and variance homogeneity (Additional file 3: Table S3). The differences in mosquito abundance for types of trapping devices were significant (*H* = 10.673, *df* = 3, *P* = 0.0136). In particular, the post hoc pairwise Wilcoxon rank sum test revealed a significant difference between BG + lure and BG + lure + CO_2_ (*P* = 0.009; Fig. 2b), suggesting that, in terms of total catch, the BG trap performed better when CO_2_ was added as a lure; no other comparisons between the total number of mosquitoes caught between the different devices were significant. The daily mean number of *Anopheles* captured was 50.8 at site 1 and 107.0 at site 2. The Wilcoxon test indicated a significantly higher number of trapped *An. messeae*/*An. daciae* for site 2 (abandoned house) compared to site 1 (farm) (*W* = 722.5, *P* = 0.0016; Fig. 2a). No significant differences were found among the four positions of the Latin square for either site (site 1, *H* = 4.244, *df* = 3, *P* = 0.236; site 2, *H* = 7.195, *df* = 3, *P* = 0.066), indicating that the observed sampling point-specific differences were likely due to chance (Fig. 2c).

There were significant differences between sites and among the four trapping devices with respect to the number of mosquitoes caught. Univariate GLMs were used to assess the effects of daily precipitation and average temperature on the number of trapped *An. messeae*/*An. daciae*. Although both variables had a significant effect [coefficient of average temperature (Coef._Tmean_) = 0.268, SE = 0.078, *P* < 0.001; coefficient of daily precipitation (Coef._Prec._) = − 0.067, SE = 0.024, *P* < 0.01], the likelihood ratio test of the negative binomial models showed that their inclusion did not significantly increase the final goodness of fit of the base model, which included just the catching sites and the device types [base model vs base model + *T*_mean_, likelihood ratio statistic (LRstat) = 1.623, *P* = 0.202; base model vs base model + Prec., LRstat = 3.736, *P* = 0.053; base model vs base model + *T*_mean_ + Prec., LRstat = 4.086, *P* = 0.130]. For this reason, Prec. and T_mean_ were not included in the subsequent analyses.

A bivariate GLM was built with a negative binomial distribution for over-dispersed count data to analyze the effect of the different trapping devices and location of captures, which were both included as fixed effects, on the number of captured *An. messeae/An. daciae.* The maximum likelihood estimate of the parameter θ for the final model is 0.684, which is indicative of over-dispersed data. Table 2 reports the exponent estimates and their 95% CIs.

**Table 2:** Incident rate ratios (i.e. exponent of the coefficient estimates), 95% confidence intervals and statistical significance for *Anopheles messeae/Anopheles daciae*

	Estimate^a^	2.5%	97.5%	*P*
Site 1	*Ref*	–	–	–
Site 2	3.420	2.046	5.722	^***^
BG + lure	2.920	1.670	5.404	^***^
BG + lure + CO_2_	14.206	8.640	25.105	^***^
CDC + CO_2_	6.253	3.677	11.361	^***^
CDC light + lure + CO_2_	8.931	5.048	16.859	^***^

For descriptions of trapping devices, see Table 1

***P < 0.001

^a^Difference in least squares means

The four trapping devices performed differently in capturing *An. messeae*/*An. daciae*. In general, a higher number of mosquitoes were caught by the BG-Sentinel and the CDC traps when they were used with the additional lures (i.e. BG + lure + CO_2_ and CDC light + lure + CO_2_) compared to the same devices used without any odor attractant. The trapping device with the highest attractiveness for *An. messeae*/*An. daciae* appeared to be the BG + lure + CO_2_ (Coef. = 14.206). Regardless of trapping device, the number of trapped *An. messeae*/*An. daciae* was 3.4 times higher at site 2 (abandoned house) than site 1 (farm). To better evaluate the significant differences between the trapping efficacies of the devices, pairwise contrasts were computed (Table 3).

**Table 3:** Contrasts between trapping device estimated marginal means after model fitting for numbers of *Anopheles messeae*/*Anopheles daciae* trapped, at a confidence level of 0.95; estimates are back-transformed from the log scale

Contrast^a^	Ratio	SE	2.5%	97.5%	*P*
BG + lure/BG + lure + CO_2_	0.206	0.075	0.101	0.420	< 0.0001
BG + lure/CDC + CO_2_	0.467	0.172	0.227	0.960	0.038
BG + lure/CDC light + lure + CO_2_	0.327	0.120	0.160	0.670	0.002
BG + lure + CO_2_/CDC + CO_2_	2.272	0.813	1.127	4.580	0.022
BG + lure + CO_2_/CDC light + lure + CO_2_	1.591	0.566	0.792	3.200	0.192
CDC + CO_2_/CDC light + lure + CO_2_	0.700	0.252	0.346	1.420	0.321

For descriptions of trapping devices, see Table 1

^a^Results are averaged for capture site

Four of the six pairwise comparisons of trap efficacies showed statistically significant differences: BG + lure vs BG + lure + CO_2_, BG + lure vs CDC + CO_2_, BG + lure vs CDC light + lure + CO_2_ and BG + lure + CO_2_ vs CDC + CO_2_. Specifically, BG + lure captured fewer mosquitoes than the other tested devices (ratio_BG+lure vs BG+lure+CO2_ = 0.206, *P* < 0.0001; ratio_BG+lure vs CDC+CO2_ = 0.467, *P* = 0.038; ratio_BG+lure vs CDC light+lure+CO2_ = 0.327, *P* = 0.002). This result can be interpreted as indicating that the addition of CO_2_ significantly improved the capability of the BG + lure device by 4.85% (i.e. 1/ratio_BG+lure vs BG+lure+CO2_); using the BG lure with CDC + CO_2_ did not lead to any significant improvement in terms of the number of captured mosquitoes (*P* = 0.321). Additional file 4: Tables S4 and S5 show the exponential estimates and the contrasts between trapping devices for the two other most represented mosquito species of the sampled population, *Cx. pipiens* and *Oc. caspius*. CDC + CO_2_ was the best trapping method for *Cx. pipiens* (Coef. = 378.52, *P* < 0.001), and the addition of lure and light did not significantly improve the number of specimens collected by it (*P* = 0.090). However the BG + lure captured 8.4 times more *Cx. pipiens* when baited with CO_2_ (1/ratio_BG+lure vs BG+lure+CO2_, *P* < 0.0001). Regarding *Oc. caspius*, both the CDC and BG traps were more effective when baited with additional lures: BG + lure + CO_2_ trapped 28.7 times more mosquitoes than BG + lure (1/ratio_BG+lure vs BG+lure+CO2_; *P* < 0.0001), and CDC light + lure + CO_2_ caught 3.2 times more mosquitoes than CDC + CO_2_ (1/ratio_CDC+CO2 vs CDC light+lure+CO2_; *P* = 0.005).

## Discussion

We report evidence from a field study that shows that both BG-Sentinel and CDC traps can be effective in sampling *An. messeae/An. daciae*. Although the BG-Sentinel trap baited with BG-Lure attracted female mosquitoes, the number of mosquitoes captured increased when more than one attractant was added. In general, traps baited with CO_2_ collected more mosquitoes. The synergistic effect of CO_2_ when used with other attractants confirms similar evidence from studies undertaken in the field [40]. In the present study, the synergistic effect of CO_2_ was particularly evident when it was used with the BG-Sentinel trap; this trap is usually only baited with an odor blend, but it caught the highest number of mosquitoes when it was also baited with CO_2_, i.e. 4.85 times more *An. messeae/An. daciae* (*P* < 0.0001) than when it was used without CO_2_*.* BG + lure + CO_2_ was also significantly (2.3 times) more effective than CDC + CO_2_ (*P* = 0.022) in collecting *An. messeae*/*An. daciae*. The BG-Sentinel trap also performed well in other trap comparison studies, which were carried out in Spain [41] and Italy [27], although for other mosquito species. Roiz et al. [41], reported that the BG trap plus BG-Lure was more effective at capturing *An. atroparvus* host-seeking and blood-fed females with or without CO_2_ than the CDC trap plus CO_2_. In the study conducted in Italy [27], four traps were tested, including the CDC trap with CO_2_ and the Biogents BG Eisenhans de Luxe trap (basically the same as BG + lure + CO_2_, but where CO_2_ release is regulated by a computer); similar to our present study, the latter was the device that caught the most *An. maculipennis* s.l. However, contrasting results were described in other studies, where the CDC trap with CO_2_ collected more *An. maculipennis* s.l. than the BG trap with BG-Lure and CO_2_, but the small number of specimens captured do not allow us to draw detailed conclusions [42, 43].

A new trap has been recently designed for the collection of *Anopheles*: the BG-Malaria trap [21]. It is very similar to BG + lure + CO_2_ used here, with minor modifications (it is an upside-down BG trap, thus the airflow is inverted), and its efficacy for sampling *Anopheles* species has been demonstrated in studies carried out in Brazil (Porto Velho, North Region) [21] and Africa (southeastern Tanzania) [44]. It is considered the most effective trap for *Anopheles* collection in endemic areas [21, 44]. Our results are consistent with those reported for traps used in these and other studies, although they were performed in areas where other malaria vector species occur [24, 45].

In our study, the CDC trap used with light and baited with lure and CO_2_ did not perform significantly better (*P* = 0.321) than the CDC trap baited with only CO_2_. This result confirmed similar outcomes in other studies showing that a source of CO_2_ is essential to increase the number of host-seeking mosquitoes caught [40, 46–48], while light and lure had no significant effect on trap efficacy [49].

Of particular interest is evaluating the performance of the CDC trap plus CO_2_ and BG-Lure, as this is the most common combination of trap and attractants used in Europe for mosquito monitoring [19, 42]. The pairwise comparisons showed that CDC + CO_2_ was superior to BG + lure (*P* = 0.038) in collecting *An. messeae*/*An. daciae* and mosquitoes of other species, and thus should be the preferred method for monitoring *Anopheles* species, although the CDC-like trap used here damaged some of the captured specimens. The key factor for better trap performance is the use of CO_2_; however, the use of CO_2_ can be a limitation due to difficulty in its supply, its cost, or the preparation of a yeast mixture [50]. Thus, a cost analysis should be done in advance regarding trap purchase, operation, and servicing to provide an indication of costs before mosquito monitoring is implemented. In our study, the purchase cost of the different traps was about the same (approximately 180 €); therefore, cost should not affect choice for either of these traps.

Manual aspiration was the most effective method for the capture of a large number of *An. messeae*/*An. daciae* and *An. maculipennis* s.s. in a short period of time during indoor collection from stables and dwellings, where these species rest. At both of the study sites, a single aspiration caught far more specimens of *An. maculipennis* s.l. (955 at site 1 and 104 at site 2) than the best-performing trapping device. However, manual aspiration is laborious and can mostly be performed only inside dwellings or shelters.

Although our results on trap performance primarily pertain to *Anopheles messeae*/*Anopheles daciae* and *Anopheles maculipennis* s.s., and are relevant to plans for further sampling of these species, they can also be used for the same purposes for other zoophilic species with similar behavior, such as *Anopheles atroparvus* and *Anopheles superpictus* [2], for which BG + lure + CO_2_ and CDC + CO_2_ are effective sampling tools. For species that are more anthropophilic, such as *Anopheles labranchiae* and *Anopheles sacharovi*, HLC remains the best method for evaluating *Anopheles* species occurrence and density, and provides additional useful information such as human biting rate [28, 51]. On the other hand, both HLC and traps baited with CO_2_ have been used successfully to collect *An. labranchiae* [6, 52].

A lower daily mean of *Anopheles* species captured by all the tested traps was observed at site 1, likely because of the presence of farm animals, which may have attracted more host-seeking mosquitoes than the traps. The influence of animal presence on catch effectiveness was observed in a previous study [53]. The difference in mosquito collection rate between the sampling sites in the present study suggests confounding factors introduced by the occurrence of competing hosts at site 1 and a subsequent reduction of their attraction to the CO_2_-baited traps. We suggest that, to perform more efficient *Anopheline* surveillance, sites should be selected that are more distant from potential hosts than those used in the present study.

*Anopheles* species were last monitored in northeast Italy in the 1990s, when *Anopheles maculipennis* s.s. was the most abundant species on the Adriatic coast and Po river delta followed by *An. atroparvus* and *An. messeae*, while *An. labranchiae* was not found [54]. Our results indicate that the species composition and distribution at the same monitoring sites have changed in the last 20 years, as *An. atroparvus* was not recorded in the present study, whereas *An. messeae/An. daciae* was the most abundant species at both sites and *An. maculipennis* s.s. was not common.

*Anopheles maculipennis* s.l. had the third highest occurrence at both sites (6.8%) after *Cx. pipiens* (62.9%) and *Oc. caspius* (27.7%). These three species represent 97.4% of all the mosquitoes collected during the study period. In general, both traps performed well in collecting these common mosquito species, but differed in their effectiveness for other less-represented species. Both the BG and CDC traps were effective in collecting *Cx. pipiens* and *Oc. caspius* when CO_2_ was added; the addition of other attractants (lure or light) only increased the number of *Oc. caspius* collected, which confirms previous observations [12, 27, 53]. For *Aedes albopictus* the BG trap has been confirmed to be the best, regardless of attractant used [27, 55, 56].

The data presented here provide researchers and field workers evidence that the tested traps, combined with certain attractants, can be used as sampling tools for *An. messeae/An. daciae*. Future work should assess the efficacy of these different trapping methods for the estimation of the abundance of the several *Anopheles* malaria vectors that occur in Europe, and the related risk of malaria transmission there.

## Conclusions

Among the trapping methods discussed in the literature for the collection of malaria vectors, most have been developed to collect host-seeking *Anopheles* females in areas with circulating malaria, whereas few studies have tested traps for potential malaria vectors in Europe. To instigate appropriate surveillance strategies to evaluate in detail the distribution and abundance of *Anopheles* species, it is necessary to identify which trap is the most suitable for their sampling. Our results show that, among the combinations of traps and attractants tested, the BG-Sentinel trap baited with BG-Lure and CO_2_ is the best for routine monitoring of host-seeking *Anopheles* mosquito species such as *An. messeae/An. daciae*. The data presented here, despite being limited to a single species of the *An. maculipennis* complex, are of interest as they indicate a standardized and useful sampling approach that needs to be further investigated for its potential to sample other potential malaria vectors in Europe. As seen in studies on other major mosquito pests, when monitoring *Anopheles* species in Europe, it is also important to consider the following: host-seeking behavior and how anthropophilic the species is, a trap’s attractiveness to the mosquitoes in relation to the natural environment, host abundance, and also cost effectiveness. This information would fill a gap in knowledge for the monitoring of potential European malaria vectors of the genus *Anopheles*, of which *An. messeae/An. daciae* is one of the most widely distributed species [57]. The possible reintroduction into Europe of *Plasmodium* parasites from endemic countries requires standardized sampling tools that allow researchers to overcome the limits to reproducibility associated with traditional approaches, such as HLC and manual aspiration. The identification of effective trapping devices for the consistent monitoring of *Anopheles* species in Europe is of major importance for malaria surveillance.

## Supplementary Information

**Additional file 1: Table S1.** Sampling sites and climatic data (temperature and precipitation) for the sampling period (trapping cycle). **Table S2.** Numbers and percentages of adults of species of the *Anopheles maculipennis* complex molecularly identified, according to collection method.

**Additional file 2: Figure S1.** DHARMa residual diagnostics plots [39] (qq-plot and residuals plotted against the predicted value) for the Poisson regression model (upper plots) and the negative binomial regression model (lower plots) for *Anopheles messeae/Anopheles daciae*. Tests for correct distribution (KS test), dispersion and outliers are shown on the plots.

**Additional file 3: Table S3.** Shapiro–Wilk test and Levene’s test to check requirements of normality and homogeneity of variance across groups, respectively.

**Additional file 4: Table S4**. Incident rate ratios (i.e. exponential of coefficient estimates), 95% CIs and significance for *Culex pipiens* and *Ochlerotatus caspius*. **Table S5**. Contrasts between trapping devices for estimated marginal means after model fitting, considering a confidence level of 0.95, for *Culex pipiens* and *Ochlerotatus caspius*. Estimates are back-transformed from the log scale.

## Data Availability

Data supporting the conclusions of this article are included within the article and its additional files. The dataset and R script generated during the study are available from the corresponding author on reasonable request.

## Abbreviations

BG trap: BG-Sentinel 2 trap
CDC trap: Centers for Disease Control and Prevention-like trap
Prec.: Total daily precipitation
Tmean: Mean daily temperature
