# Supplementary material for: Comparative efficacy of BG-Sentinel 2 and CDC-like mosquito traps for monitoring potential malaria vectors in Europe
Source: Parasit Vectors. 2022 May 7;15:160. doi: 10.1186/s13071-022-05285-9 (PMC9077833; doi:10.1186/s13071-022-05285-9)
Supplement: Supplementary file 1 — Additional file 1: Table S1. Sampling sites and climatic data (temperature and precipitation) for the sampling period (trapping cycle). Table S2. Numbers and percentages of adults of species of the Anopheles maculipennis complex molecularly identified, according to collection method. [file 13071_2022_5285_MOESM1_ESM.docx]

**Additional file 1.**

Table S1: Sampling sites and climatic data (temperature and precipitation) during the sampling period (trapping cycle).

| Site | Description (aggregated biotope) | Trapping cycle | Mean temperature (°C) during sampling period (95% CI) | Total precipitations (mm) during sampling period (no. rainy days) |
| --- | --- | --- | --- | --- |
| 1 | farm  (agricultural) | 09-12 Jul 2019 | 23.450 (23.050-23.850) | 0.4 (1) |
|  |  | 06-09 Aug 2019 | 25.500 (23.458-27.542) | 13.8 (2) |
|  |  | 03-06 Sept 2019 | 20.825 (17.553-24.097) | 37.2 (1) |
| 2 | abandoned house  (agricultural) | 02-05 Jul 2019 | 26.050 (23.866-28.234) | 5.8 (1) |
|  |  | 30 Jul-02 Aug 2019 | 25.075 (22.588-27.562) | 9.4 (1) |
|  |  | 27-30 Aug 2019 | 25.150 (24.622-25.678) | 0.0 (0) |

95% CI: Confidence interval

Table S2: Number and percentage of *An. maculipennis* complex species molecularly identified according to collection method.

|  | Site 1 | | Site 2 |
| --- | --- | --- | --- |
|  | *An. messeae*/*daciae* (%) | *An. maculipennis* s.s. (%) | *An. messeae*/*daciae* (%) |
| BG lure | 19 (18.4%) | 1 (12.5%) | 33 (23.1%) |
| BG lure+CO_2_ | 33 (32.0%) | 4 (50.0%) | 37 (25.9%) |
| CDC+CO_2_ | 24 (23.2%) | 2 (25.0%) | 36 (25.1%) |
| CDC light+lure+CO_2_ | 27 (26.2%) | 1 (12.5%) | 37 (25.9%) |
| Total | 103 (92.8%) | 8 (7.2%) | 143 (100.0%) |
