# Supplementary material for: Comparative efficacy of BG-Sentinel 2 and CDC-like mosquito traps for monitoring potential malaria vectors in Europe
Source: Parasit Vectors. 2022 May 7;15:160. doi: 10.1186/s13071-022-05285-9 (PMC9077833; doi:10.1186/s13071-022-05285-9)

**Additional file 2.**

Figure S1: DHARMa residual diagnostics plots [39] (qq-plot and residuals against the predicted value) for the Poisson regression model (upper plots) and the negative binomial regression model (lower plots) for *An. messeae/daciae*. Tests for correct distribution (KS test), dispersion and outliers are included in the plots.


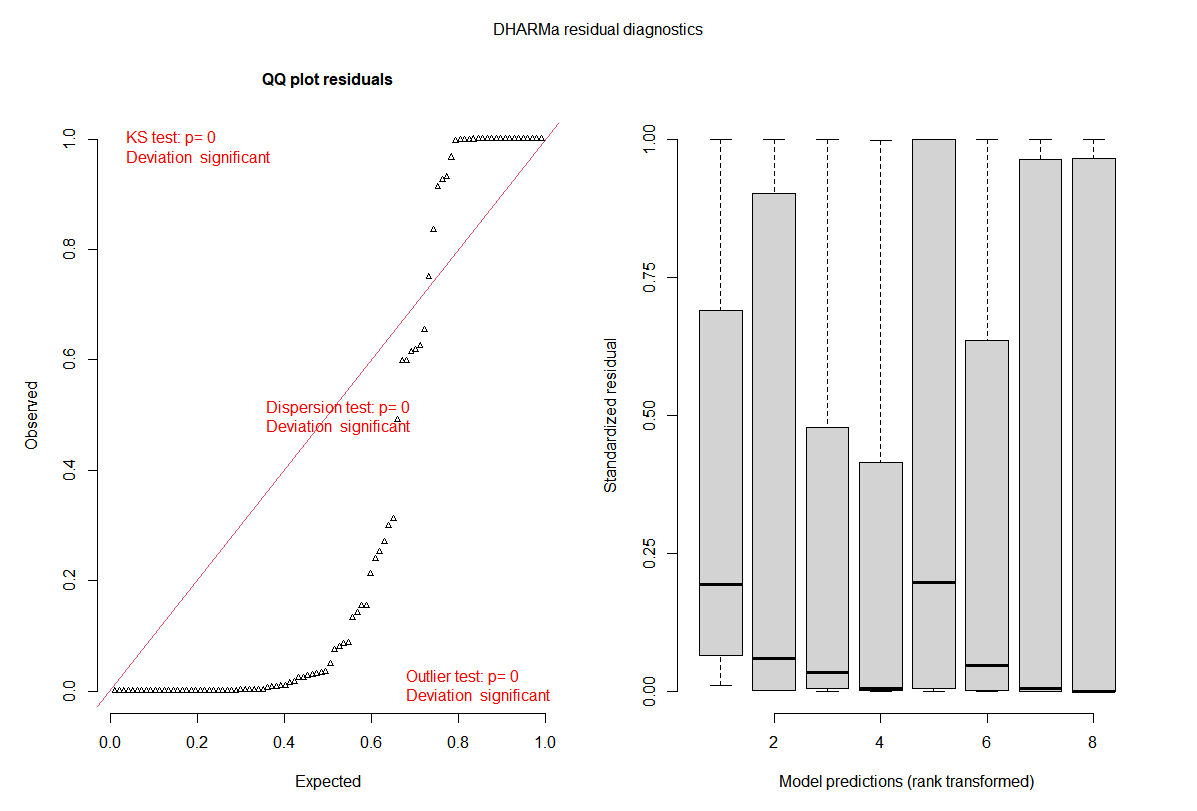


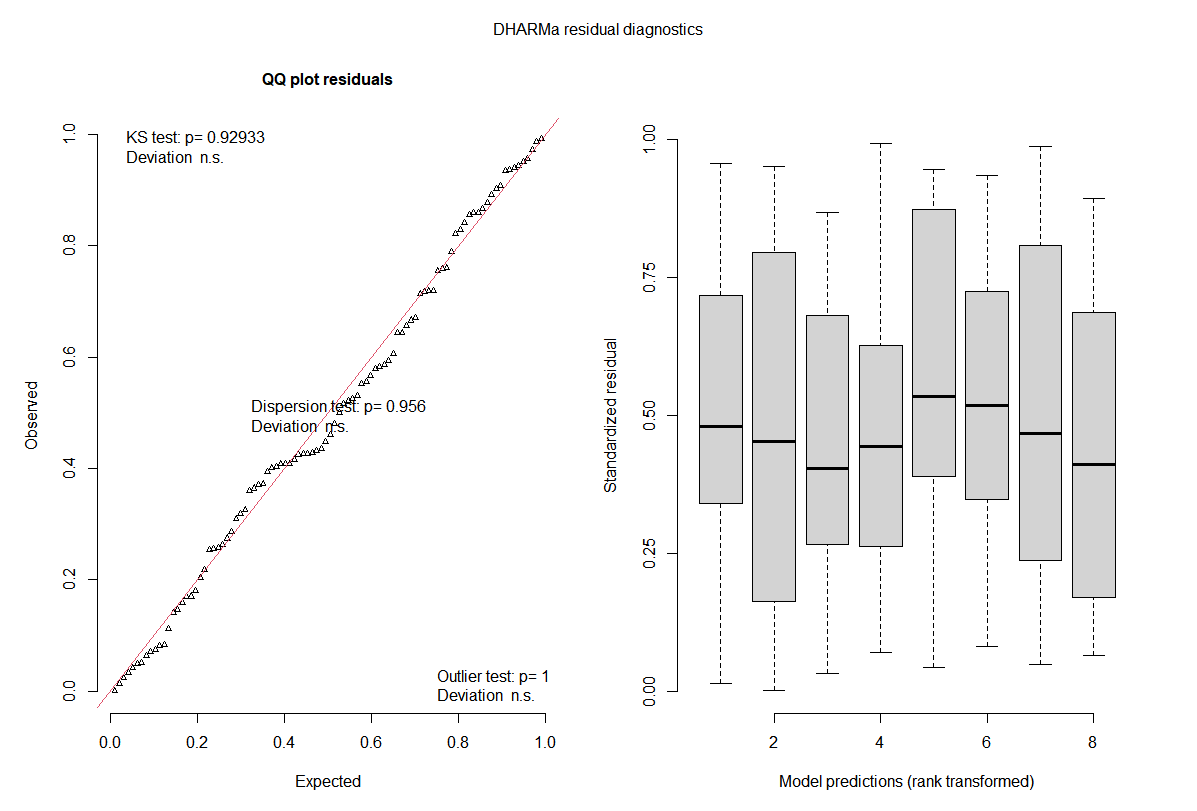

Supplement: Supplementary file 2 — Additional file 2: Figure S1. DHARMa residual diagnostics plots [39] (qq-plot and residuals plotted against the predicted value) for the Poisson regression model (upper plots) and the negative binomial regression model (lower plots) for Anopheles messeae/Anopheles daciae. Tests for correct distribution (KS test), dispersion and outliers are shown on the plots. [file 13071_2022_5285_MOESM2_ESM.docx]
