# Supplementary material for: Comparative efficacy of BG-Sentinel 2 and CDC-like mosquito traps for monitoring potential malaria vectors in Europe
Source: Parasit Vectors. 2022 May 7;15:160. doi: 10.1186/s13071-022-05285-9 (PMC9077833; doi:10.1186/s13071-022-05285-9)
Supplement: Supplementary file 3 — Additional file 3: Table S3. Shapiro–Wilk test and Levene’s test to check requirements of normality and homogeneity of variance across groups, respectively. [file 13071_2022_5285_MOESM3_ESM.docx]

**Additional file 3.**

Table S3: Shapiro-Wilk test and Levene's test to check requirements of normality and homogeneity of variance across groups, respectively.

|  | Shapiro-Wilk Test^a^ | Levene's Test^a^ |
| --- | --- | --- |
| Type of trapping device | W = 0.726  *P* < 0.0001 | F_(3,92)_ = 2.592  *P* = 0.0574 |
| Site | W = 0.749  *P* < 0.0001 | F_(1,94)_ = 9.841  *P* = 0.0023 |
| Latin square trap location | Site 1  W = 0.826  *P* < 0.0001  Site 2  W = 0.868  *P* < 0.0001 | Site 1  F_(3,44)_ = 2.193  *P* = 0.1023  Site 2  F_(3,44)_ = 3.100  *P* = 0.0362 |

^a^*P* values < 0.05 lead to the rejection of the null hypothesis associated with the normality of data distribution and homogeneity of variances.
