# Supplementary material for: Comparative efficacy of BG-Sentinel 2 and CDC-like mosquito traps for monitoring potential malaria vectors in Europe
Source: Parasit Vectors. 2022 May 7;15:160. doi: 10.1186/s13071-022-05285-9 (PMC9077833; doi:10.1186/s13071-022-05285-9)
Supplement: Supplementary file 4 — Additional file 4: Table S4. Incident rate ratios (i.e. exponential of coefficient estimates), 95% CIs and significance for Culex pipiens and Ochlerotatus caspius. Table S5. Contrasts between trapping devices for estimated marginal means after model fitting, considering a confidence level of 0.95, for Culex pipiens and Ochlerotatus caspius. Estimates are back-transformed from the log scale. [file 13071_2022_5285_MOESM4_ESM.docx]

**Additional file 4**

Table S4**.** Incident rate ratios (i.e., exponential of coefficient estimates), 95% confidence intervals and significance, for *Cx. pipiens* and *Oc. caspius*.

|  | *Cx. pipiens* | | | | *Oc. caspius* | | | | |
| --- | --- | --- | --- | --- | --- | --- | --- | --- | --- |
|  | Estimate | 2.5 % | 97.5 % | *P* | Estimate | 2.5 % | 97.5 % | *P* | |
| Site 1 | *ref.* | - | - | - | *ref.* | - | - | - |  |
| Site 2 | 0.809 | 0.468 | 1.397 |  | 2.130 | 1.164 | 3.937 | ^**^ |  |
| BG+lure | 17.909 | 10.442 | 33.450 | ^***^ | 2.584 | 1.242 | 5.749 | ^**^ |  |
| BG+lure+CO_2_ | 150.629 | 90.381 | 274.269 | ^***^ | 74.231 | 42.640 | 143.201 | ^***^ |  |
| CDC+CO_2_ | 378.520 | 211.978 | 736.138 | ^***^ | 29.637 | 16.535 | 58.745 | ^***^ |  |
| CDC light+lure+CO_2_ | 199.738 | 110.772 | 391.770 | ^***^ | 94.062 | 52.826 | 185.510 | ^***^ |  |

Estimate: differences in least squares means; *P*: *P* value; ***: *P* < 0.001; **: *P* < 0.01

Table S5. Contrasts between trapping devices estimated marginal means after model fitting, considering a confidence level of 0.95, for *Cx. pipiens* and *Oc. caspius*. Estimates are back-transformed from the log scale.

|  | *Cx. pipiens* | | | | | *Oc. caspius* | | | | |
| --- | --- | --- | --- | --- | --- | --- | --- | --- | --- | --- |
| Contrast^a^ | Ratio | SE | 2.5 % | 97.5 % | *P* | Ratio | SE | 2.5 % | 97.5 % | *P* |
| BG lure / BG lure+CO_2_ | 0.119 | 0.045 | 0.056 | 0.250 | < 0.0001 | 0.035 | 0.015 | 0.015 | 0.080 | < 0.0001 |
| BG lure / CDC+CO_2_ | 0.047 | 0.018 | 0.023 | 0.100 | < 0.0001 | 0.087 | 0.037 | 0.038 | 0.199 | < 0.0001 |
| BG lure / CDC light+lure+CO_2_ | 0.090 | 0.034 | 0.043 | 0.189 | < 0.0001 | 0.028 | 0.012 | 0.012 | 0.063 | < 0.0001 |
| BG lure+CO_2_ / CDC+CO_2_ | 0.398 | 0.150 | 0.190 | 0.834 | 0.015 | 2.505 | 1.022 | 1.125 | 5.575 | 0.025 |
| BG lure+CO_2_ / CDC light+lure+CO_2_ | 0.754 | 0.285 | 0.360 | 1.580 | 0.455 | 0.789 | 0.322 | 0.355 | 1.754 | 0.561 |
| CDC+CO_2_ / CDC light+lure+CO_2_ | 1.895 | 0.715 | 0.905 | 3.968 | 0.090 | 0.315 | 0.129 | 0.142 | 0.701 | 0.005 |

^a^ Results are averaged over the levels of catching site.
